# Editorial: Technological Advances in Microbiological Risk Assessment

**DOI:** 10.3389/fmicb.2022.872879

**Published:** 2022-03-17

**Authors:** Jun Wang, Biao Suo, Fereidoun Forghani, Gongliang Zhang, Bruce Applegate

**Affiliations:** ^1^College of Food Science and Engineering, Qingdao Agricultural University, Qingdao, China; ^2^College of Food Science and Technology, Henan Agricultural University, Zhengzhou, China; ^3^Molecular Epidemiology, Inc., Lake Forest Park, WA, United States; ^4^School of Food Science and Technology, Dalian Polytechnic University, Dalian, China; ^5^Department of Food Science, Purdue University, West Lafayette, IN, United States; ^6^Department of Biological Sciences, Purdue University, West Lafayette, IN, United States

**Keywords:** microbiological risk assessment, predictive microbiology, rapid detection, growth model, inactivation model

This Research Topic focused on current advances of research related to microbiological risk assessment (MRA). To minimize the adverse impact of foodborne pathogens on human health, MRA has been regarded as a structured and effective approach to improve food control systems and evaluate microbial risks. Generally, MRA is conducted in response to well defined risk management questions, requiring huge available data input to provide relevant results. In response to recent scientific and technical advances, and public demands, 10 articles were collected according to the objectives of this topic and could be divided into the following four aspects:

## Improving Exposure Assessment step of MRA

In MRA, predictive models play an important role for exposure assessment of foodborne pathogens to describe the microbial response over time and the growth dynamics affected by environmental conditions during the food chain from farm to table. Hiura et al. developed a Bayesian statistical modeling based on a generalized linear model (GLM) to fit observed bacterial inactivation data and growth data for *Bacillus simplex* and *Listeria monocytogenes*, respectively. Accordingly, the bacterial inactivation or growth, considering variability and uncertainty was simulated. The developed models enable a more explicit illustration of the variation in bacterial behavior *via* probability distributions. The novel method could clearly explain the variability and uncertainty in bacterial population behavior and could provide as useful information for risk assessment related to food borne pathogens. In another study by Zhou et al. the Weibull model was designed to evaluate the effectiveness of a newly developed 360-degree radiation thermosonication system (TS) in inactivating the *Staphylococcus aureus* in milk. In addition, the Bigelow and Log-linear model with tail were successfully used for describing the thermal inactivation kinetics of *Listeria monocytogenes* under mild heat, lactic acid, benzalkonium chloride, and nisin treatments, while the model- derived extended lag time of the survivors can be used to evaluate the cell growth kinetics following the treatments (Fang et al.).

## Supporting MRA in Hazard Identification

In hazard identification, food contamination surveillance data, together with product/process evaluations needs to be collected, appraised, and interpreted to aid the identification of hazard–food combinations. In the study by Yan et al. the serotypes, MLST, and cgMLST of *Salmonella enterica* isolates from different sources in nine provinces in China from 2004 to 2019 were examined and used to investigate their phenotyping and genotyping diversities and genetic relationships. This article clarifies the temporal and spatial distribution characteristics of phenotyping and genotyping diversity of *S. enterica* isolates in China in the recent 16 years, which could provide valuable information for prevention and control of *Salmonella* in China with strain resources and genetic information. In another study by Qu et al. the prevalence of *B. cereus* in lettuce and farm environments distributed in China was investigated to determine the possible transmission of *B. cereus* on lettuce farms in China and its enterotoxicity. The results showed that soil and pesticides are the main sources of *B. cereus* on lettuce farms in China, and the possible transmission routes are as follows: soil-lettuce, manure-lettuce, pesticide-lettuce, manure-soil-lettuce, and water-manure-soil-lettuce. Furthermore, the *B. cereus* isolates, whether from lettuce or the environment, pose a potential risk to health. Liu et al. focused on the investigation of *Escherichia coli* strains isolated from raw milk of dairy cattle in Northern China and their antimicrobial susceptibility and essential virulence genes. The importance of this topic comes from the fact that *E. coli* is commonly associated with animals and is a major cause of toxic mastitis in dairy cows. Results obtained in the study showed that 34.4% of the samples were positive for *E. coli*, and that among the positive samples, several of them were harboring toxic genes and/or showed antimicrobial resistance. This aligned well with other literature emphasizing that antimicrobial resistance should be of concern to the public health authorities and in this particular case, that antibiotics should be cautiously used for the treatment of *E. coli* caused mastitis in dairy cows.

## Improving MRA and Prevention Approaches

Bahk and Lee developed a user-friendly Microbial-MLE Tool, which can be easily used without requiring complex mathematical knowledge of MLE using an Excel spreadsheet. The tool, which is designated to adjust log-normal distributions to observed counts and implemented for food microbial censored data, would provide an accessible and easily comprehensible means for performing MLE and useful calculation to improve the outcome of MRA.

## Rapid Detection or Approaches Reducing Risk

Effective and rapid detection of foodborne pathogens based on emerging technologies is critical for reliably assessing the pathogenic factors and reducing microbial risk. The review by Huang et al. explained how the aptasensors have been applied to risk assessment in foodborne pathogens using *Staphylococcus aureus* as a representation. The review concluded that the aptasensors have a good competitiveness for using as a tool for risk assessment of foodborne pathogens, in terms of time, sensitivity, specificity, and cost, especially with the developments of nanomaterials and portable detection instruments in future. Mustafa et al. assessed the heavy metal resistance in *Salmonella* Typhimurium and its association with disinfectant and antibiotic resistance. The research conclusion was that excessive use of metals and disinfectants as feed additive in animal care may have the potential to promote antibiotic resistance through co-selection and maintain and promote antibiotic resistance even in the absence of antibiotics. Xu and Zhu investigated the positive effects of complete replacement of nitrite with a *Lactobacillus fermentum* on the quality and safety of Chinese fermented sausages, and evaluated the risk of this strain. The results showed that replacing nitrite completely with the *L. fermentum* strain could be a potential strategy to produce healthier and safer acceptable sausages through decreasing the risk of nitrite and improving nutrition and quality of the sausages.

By compiling these 10 articles into this topic, the advances in MRA including development of growth/inactivation model, the rapid detection method, prevalence and molecular characterization of foodborne pathogens from different matrices, as well as emerging technologies on the inactivation of foodborne pathogens were covered, providing useful information for the target audience.

## Author Contributions

JW: conceptualization, writing the original draft, funding acquisition, writing—review, and editing. BS: writing the original draft, writing—review, and editing. FF: writing the original draft, writing—review, and editing. GZ: writing—review and editing. BA: conceptualization, writing—review, and editing. All authors approved the submitted version.

## Funding

Research in the JW lab was supported by the Grant from China National Center for Food Safety Risk Assessment (20210349-6602421216), BS was supported by Natural Science Foundation of Henan Province (222300420455), and National Risk Assessment Major Special Project of Milk Product Quality and Safety (GJFP2019027).

## Conflict of Interest

FF was employed by the company IEH Laboratories and Consulting Group, United States. The remaining authors declare that the research was conducted in the absence of any commercial or financial relationships that could be construed as a potential conflict of interest.

## Publisher's Note

All claims expressed in this article are solely those of the authors and do not necessarily represent those of their affiliated organizations, or those of the publisher, the editors and the reviewers. Any product that may be evaluated in this article, or claim that may be made by its manufacturer, is not guaranteed or endorsed by the publisher.

